# Positive Feedbacks in Seagrass Ecosystems – Evidence from Large-Scale Empirical Data

**DOI:** 10.1371/journal.pone.0016504

**Published:** 2011-01-24

**Authors:** Tjisse van der Heide, Egbert H. van Nes, Marieke M. van Katwijk, Han Olff, Alfons J. P. Smolders

**Affiliations:** 1 Community and Conservation Ecology Group, Centre for Ecological and Evolutionary Studies (CEES), Groningen University, Haren, The Netherlands; 2 Aquatic Ecology and Water Quality Management Group, Department of Environmental Sciences, Wageningen University, Wageningen, The Netherlands; 3 Department of Environmental Science, Institute for Wetland and Water Research, Faculty of Science, Radboud University Nijmegen, Nijmegen, The Netherlands; 4 Department of Aquatic Ecology and Environmental Biology, Institute for Water and Wetland Research, Faculty of Science, Radboud University Nijmegen, Nijmegen, The Netherlands; Dalhousie University, Canada

## Abstract

Positive feedbacks cause a nonlinear response of ecosystems to environmental change and may even cause bistability. Even though the importance of feedback mechanisms has been demonstrated for many types of ecosystems, their identification and quantification is still difficult. Here, we investigated whether positive feedbacks between seagrasses and light conditions are likely in seagrass ecosystems dominated by the temperate seagrass *Zostera marina*. We applied a combination of multiple linear regression and structural equation modeling (SEM) on a dataset containing 83 sites scattered across Western Europe. Results confirmed that a positive feedback between sediment conditions, light conditions and seagrass density is likely to exist in seagrass ecosystems. This feedback indicated that seagrasses are able to trap and stabilize suspended sediments, which in turn improves water clarity and seagrass growth conditions. Furthermore, our analyses demonstrated that effects of eutrophication on light conditions, as indicated by surface water total nitrogen, were on average at least as important as sediment conditions. This suggests that in general, eutrophication might be the most important factor controlling seagrasses in sheltered estuaries, while the seagrass-sediment-light feedback is a dominant mechanism in more exposed areas. Our study demonstrates the potentials of SEM to identify and quantify positive feedbacks mechanisms for ecosystems and other complex systems.

## Introduction

Ecosystems are often characterized by complex biotic and abiotic interactions. Positive feedbacks are a type of interaction that is especially relevant because such mechanisms typically cause a complex, nonlinear response of ecosystems to environmental changes. Moreover, if feedbacks are strong enough, theory suggests that these self-amplifying mechanisms may cause alternative stable states [1], [2]. In such systems, small changes or disturbances may push the ecosystem beyond a critical threshold, causing a sudden shift to an alternative state [1], [3]. Despite the fact that the relevance of these mechanisms has been demonstrated for a wide range of terrestrial, freshwater and marine ecosystems [1], [3], identification of positive feedbacks and quantification of their importance for any particular type of ecosystem (e.g. lakes, salt-marshes, deserts) still remains difficult. Large-scale (cross-ecosystem) datasets can provide hints for bistability – for instance if there are sudden jumps in time series or if the data is bimodally distributed [2]. These types of analyses, however, do not provide any mechanistic explanation for observed trends or distributions. Experiments, on the other hand, can provide convincing evidence for alternative stable states and the underlying mechanisms [2], but cannot indicate whether the identified mechanisms are relevant for the full-scale complexity in the field situation.

Seagrass meadows are a type of ecosystem where positive feedbacks and bistability have been suggested both from small-scale experimental and modeling studies [4], [5], [6]. These ecosystems are of great importance to many of the World's coastal areas because they enhance biodiversity and provide important ecosystem services (e.g. storm buffering, nutrient cycling, fisheries) [7], [8], [9]. Over the last century, however, seagrasses have become increasingly affected by human activities, which cause severe declines that are often characterized by sudden losses [8], [9]. Seagrasses are ecosystem engineers, in the sense that they significantly modify the abiotic environment of their ecosystem [10]. For instance, seagrasses relieve hydrodynamic stress by attenuating currents and waves [11], [12], [13] and improve light conditions by reducing suspended sediment levels [14], [15], [16], [17], [18] and nutrient concentrations [5], [19]. Still, because seagrass ecosystems are typically exposed to multiple environmental stressors (e.g. light limitation, hydrodynamics, salinity) and human disturbances (e.g. siltation events, eutrophication, dredging activities) [8], assessing the importance of positive feedbacks in the field situation is very difficult.

In this study, we quantified a positive feedback between seagrasses and light conditions – probably the most important environmental variable influencing seagrass [20], [21] – in systems dominated by the temperate seagrass *Zostera marina*. For this analysis, we used a combination of multiple linear regression and path analysis by structural equation modeling (SEM) [22] on a large-scale dataset containing 83 sampling locations scattered all over Western Europe. We examined the relations between nutrients, sediments, light conditions and seagrass which allowed us to identify a generic positive feedback mechanism between sediment, light conditions and seagrass in this type of ecosystem and quantify its importance relative to other factors influencing light and seagrass growth.

## Methods

### Data collection

**Figure 1:**
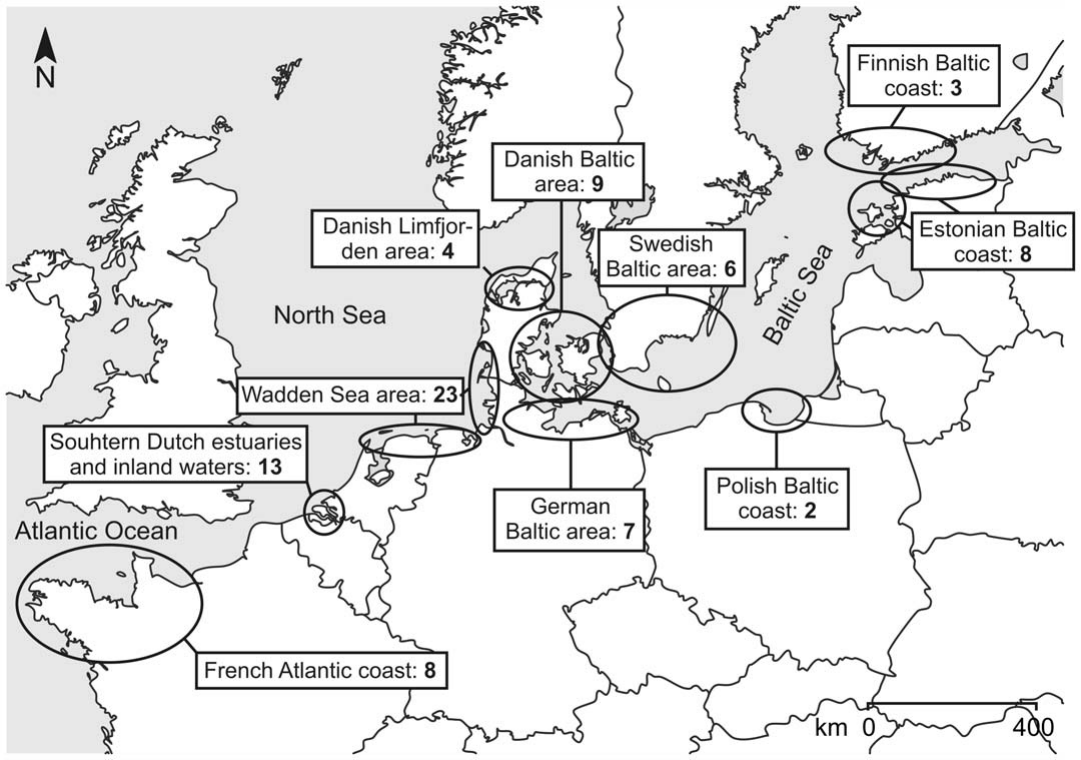
General overview of the geographical setting of the 83 sampled sites. All sites are located in regions where seagrass is present or has been present in the past.

Data were collected in several regions across Western Europe (Fig. 1), where seagrasses are present or were historically present. Within these regions, sites were randomly selected. Each site was sampled once in the growing season (May to September) of 2005. Water depth of the sampling locations varied between 0.5 m above to 5 m below the mean water level. At each site, we recorded the total area covered by seagrass patches and estimated the average shoot density (sh m^−2^) within patches by counting shoots within a 0.25×0.25 m stainless steel frame (3–5 randomly selected replicates per site). From these observations, we estimated the mean shoot density by multiplying the standardized cover by shoot density. We used the light attenuation coefficient of the water column as a proxy for the light conditions at each site. Light attenuation (m^−1^) was measured in PAR (400–700 nm) using a quantum light meter (Li-192, Li-Cor). Next, we sampled and pooled 3 replicates of surface water taken approximately 25 to 50 m apart. From these samples, we determined total nitrogen and total phosphorus, which were used as indicators for nutrient availability and growth potential of phytoplankton. Finally, we sampled and pooled 3 replicates of sediment (top 10 cm) from bare areas, i.e. areas outside seagrass meadows (at least 25 m from the edge of the meadow). We used grain size distribution of these samples as a proxy for suspendability of the sediment. We specifically chose to sample sediments outside the meadows, because these are susceptible to erosion, while sediments inside the seagrass beds are trapped and stabilized [14], [17], [23].

### Chemical analyses

Total nitrogen and total phosphorus (µmol l^−1^) in the surface water were measured as nitrate and ortho-phosphate after digestion with persulfate [24]. The concentration of ortho-phosphate in the digested samples was determined colorimetrically using ammonium-molybdate [25]. Nitrate was determined by sulphanilamide after reduction to nitrite in a cadmium column [26]. Grain size distribution of the sediment was measured on freeze-dried samples by laser diffraction on a Beckman Coulter particle size analyzer. We determined D10, D50 and D90 that describe the grain size (µm) at which 10%, 50%, and 90%, respectively of the volumetric fraction is smaller. All devices were calibrated according to standardized procedures provided by the manufacturers. For all analyses quality assurance measures included blanks, replicate analyses and matrix spikes. Recoveries from matrix spikes ranged from 95% to 107%. Repeated analyses did not reveal differences greater than 5%.

### Data analysis

To obtain normal distribution, we applied square root transformation (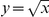) to average shoot density. All other variables that were not normally distributed were either logarithmically (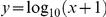) or reciprocally (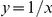) transformed. To test which of the surface water and sediment variables influenced light attenuation and should therefore be included in the structural equation model, we first examined the relationship between light and these variables by multiple linear regression (MLR) with stepwise backward elimination in the software package SPSS (v18, SPSS inc.). The criterion for the probability of F-to-exclude was >0.05. Subsequently, we tested for correlation between included variables using single linear regression.

**Figure 2:**
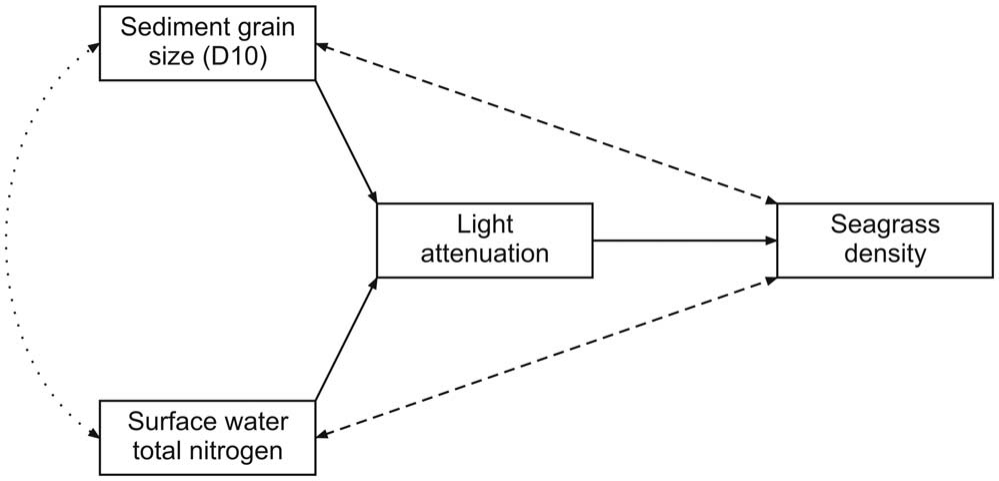
Diagram of a conceptual structural equation (SE) model describing possible relationships between sediment grain size (D10), total nitrogen, light attenuation and seagrass density. Bidirectional dashed arrow lines depict relationships of which the causality and relevance are not sure. We also tested for covariance between residuals of total nitrogen and sediment D10, which is indicated by the dotted arrow line.

Subsequently, we constructed a structural equation (SEM) model in the software package AMOS (v18, SPSS inc.) that included seagrass density, light attenuation and the variables selected by the MLR procedure: D10 and total nitrogen (see paragraph 1 of the results section). Figure 2 shows the conceptual model describing possible relationships between *D10*, *total nitrogen*, *light attenuation* and *seagrass density*. Relevance and causality of the relationships between *seagrass density – sediment grain size* and *seagrass density – total nitrogen* are not obvious a priori. Seagrasses might influence surface water total nitrogen through direct uptake of nitrate or ammonia [5], [19]. However, total nitrogen might also influence seagrass density directly, for instance by means of ammonia toxicity [5], [27]. Similarly, seagrasses may increase the coarseness of the sediment outside the bed by trapping small particles inside the meadow [6], [17], [18], [23], [28]. On the other hand, sediment grain size outside the bed might also correlate with seagrass density because of its general characteristics (e.g. nutrient content, organic matter) at the sampling site. Finally, possible relationships for both *seagrass density – total nitrogen* and *seagrass density – sediment grain size* could even be bidirectional, causing direct positive feedbacks. For instance, in eutrophicated systems, high seagrass density could reduce ammonium levels, which would in turn enhance seagrass growth again [5]. In contrast, trapping of small particles from the surrounding area by seagrass could cause a positive feedback in nutrient poor systems because this mechanism would increase nutrient availability in the sediment [17], [29]. To elucidate the importance of these possible relations, we constructed a model with bidirectional relationships for these variables. Next, we tested all possible combinations of relationships between *seagrass density – sediment grain size* and *seagrass density – total nitrogen* using the “specification search” option in AMOS and ranked the models according to Akaike's Information Criterion (AIC) and the χ^2^ test. We also tested for possible covariance between residuals of *total nitrogen* and *sediment grain size*. Unidentifiable and unstable models (i.e. models with a stability index outside the -1 to 1 range) as well as models with non-significant relations (i.e. coefficients with *p*>0.05) were excluded from the results.

## Results

Our 83-site database included 34 locations with *Zostera marina* present. Average shoot densities at these sites varied from less than 1 to over 740 sh/m^2^ (mean: 210 (±221 SD) sh/m^2^). Of all variables (D10, D50, D90, total nitrogen, total phosphorus) only D10 – the grain size parameter describing the smallest fraction – and total nitrogen were included by the MLR procedure to explain light attenuation (Table 1). The two included variables were able to explain 61% of the variance in light attenuation. Linear regression of total nitrogen versus D10 demonstrated that there was no significant correlation between these two variables (*F* = 3.919; *p* = 0.051; *R^2^* = 0.046).

**Table 1:** Results from the multiple linear regression analysis with stepwise backward selection of explanatory variables.

General model statistics			
*R^2^*	*Adj. R^2^*	*F*	*p*
0.606	0.596	61.492	<0.000

Parameter statistics			
	*Coefficient*	*SE*	*p*
Constant	2.606	0.366	<0.000
Sediment D10	0.009	0.001	<0.000
Total nitrogen	−1.338	0.204	<0.000

D10 (µm) was untransformed. Total nitrogen (µmol l^−1^), total phosphorus (µmol l^−1^), D50 (µm) and D90 (µm) were logarithmically transformed. Light attenuation (m^−1^) was reciprocally transformed.

Total phosphorus, D50 and D90 were eliminated from the model by the selection procedure. Thus, only sediment grain size D10 and surface water total nitrogen were included to describe the dependent variable light attenuation.

**Figure 3:**
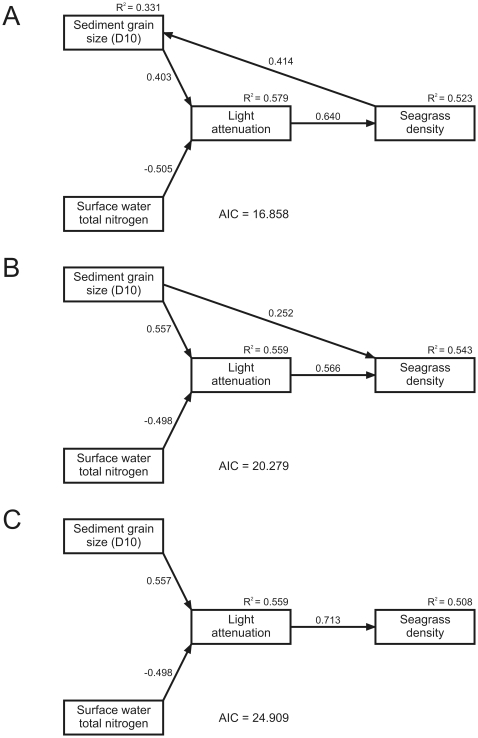
Diagram of the three stable and significant structural equation models. **A**) Model 1 provided the best fit to the data. It includes a positive feedback loop between sediment grain size (D10), light attenuation and seagrass density. **B**) The second best model (2) describes the relation between seagrass and sediment grain size in the opposite direction compared to model 1. **C**) The third, lowest-ranking model (3) was the simplest of the three model and did not include a direct relation between seagrass and sediment grain size. Note that light attenuation was reciprocally transformed and that the effect of nitrogen is therefore negative, while the effect of sediment D10 is positive (higher value for light attenuation  =  increased water clarity). Values above the arrow lines depict the standardized regression weights.

The SEM specification search procedure yielded only three stable models with significant coefficients (Fig. 3 and Table 2). All three models demonstrate a significant positive effect of sediment grain size (D10) and a negative effect of total nitrogen on the reciprocally transformed light attenuation (higher value  =  increased water clarity). None of the models showed significant covariance between residuals of total nitrogen and sediment grain size. Light conditions had a strong positive effect on seagrass density. Of the three models, the simplest one, that is without direct *seagrass – total nitrogen* and *seagrass – sediment* interactions, ranked third. The two highest-ranking models had a single direct relationship (but in opposite directions) between seagrass density and sediment D10, but not with total nitrogen. The highest-ranking model included a positive feedback between sediment grain size, light conditions and seagrass density. Compared to the second model, the first-ranked model demonstrated lower AIC and Chi-square values, a higher probability level and stronger significance for the regression weight between seagrass density and sediment D10 (Table 2). Despite the fact that in model 2, sediment D10 was added as direct explanatory variable for seagrass density, it was able to explain only slightly more of the variance in seagrass density (54.3%) compared to model 1 and 3 that explained 52.3% and 50.8% respectively. Additionally, results showed that the standardized indirect effect of sediment D10 on seagrass density (0.315) was more important that its direct effect (0.252). Due to the positive feedback loop in model 1, it was able to explain 33.1% of all variance in sediment D10, while *R^2^* of light attenuation also improved slightly from 0.559 to 0.579. The standardized indirect effect of seagrass density on light in model 1 was 0.187, i.e. an increase of 1× standard deviation (SD) in seagrass density resulted in an increase of 0.187 SD in the reciprocally transformed light attenuation. The standardized total ( =  indirect + direct) effect of seagrass density on itself was 0.119, while total effects of total nitrogen, sediment D10 and light attenuation on seagrass density were −0.362, 0.288 and 0.716 respectively.

**Table 2:** Results from the structural equation modeling (SEM) specification search.

	Model 1	Model 2	Model 3
	*Model fit summary*		
*AIC* (# parameters)	16.858 (8)	20.279 (8)	24.909 (7)
*χ^2^*	0.858	4.279	10.909
*df*	2	2	3
*Probability level*	0.651	0.118	0.012
	*Squared multiple correlations (R^2^)*		
Light attenuation	0.579	0.559	0.559
Seagrass density	0.523	0.543	0.508
Sediment D10	0.331	–	–
	*Standardized regression weights (p-value)*		
Total nitrogen ≫ Light attenuation	−0.505 (<0.000)	−0.498 (<0.000)	−0.498 (<0.000)
Sediment D10 ≫ Light attenuation	0.403 (<0.000)	0.557 (<0.000)	0.557 (<0.000)
Light attenuation ≫ Seagrass density	0.640 (<0.000)	0.566 (<0.000)	0.713 (<0.000)
Seagrass density ≫ Sediment D10	0.414 (<0.000)	–	–
Sediment D10 ≫ Seagrass density	–	0.252 (0.005)	–

Seagrass density (sh m^−2^) was square root transformed, light attenuation (m^−1^) was reciprocally transformed, D10 (µm) was untransformed and total nitrogen (µmol l^−1^) was logarithmically transformed.

We found no significant direct relation between total nitrogen and seagrass density. For the relation between seagrass density and sediment D10, the relation was strongest when causality was modeled from seagrass density to sediment D10. Note that light attenuation was reciprocally transformed and that effect of nitrogen is therefore negative, while the effect of sediment D10 is positive (higher value for light attenuation  =  increased water clarity).

## Discussion

Despite the fact that the importance of positive feedback mechanisms is recognized for many types of ecosystems (e.g. lakes, deserts, coral reefs, salt marshes, seagrasses), identification or quantification of the relative importance of feedbacks in any particular kind of ecosystem is typically difficult [1], [2]. By using a combination of multiple linear regression and structural equation modeling, we were able to detect and quantify a positive feedback, which indicated that seagrasses are able to trap and stabilize sufficient amounts of suspended sediments to improve water clarity and thus seagrass growth conditions.

Since our dataset covers a broad geographical area (i.e. Western Europe) with many different types of systems (i.e. open ocean conditions, sheltered estuaries, brackish inland waters) our results suggest that the identified feedback is generic for seagrass ecosystems. Nevertheless, relevance of this feedback probably varies strongly depending on the local abiotic environment. In systems like for instance the Wadden Sea, where turbidity is sediment-dominated, trapping and stabilization of silt particles by seagrass beds may be very important to sustain adequate growing conditions for seagrass itself. In such ecosystems, this positive feedback could potentially lead to alternative stable states [6]. Overall, however, eutrophication, as indicated by total nitrogen, is at least as important as sediment conditions in controlling water clarity and seagrass density. During our survey, estuaries dominated by high nutrient levels (and phytoplankton) were mainly found in the Baltic Sea, the Limfjorden and Dutch brackish inland waters. It appears that eutrophication can result in severe seagrass decline in these systems. However, because these systems are typically sheltered, hydrodynamics are generally low and sediment resuspension is therefore often limited. Hence, the risk of sudden collapse due to disturbance of the light-seagrass-sediment feedback might be less high in these systems compared to for instance the Wadden Sea area. Furthermore, it should be noted, that we found no evidence of any direct feedback between seagrass and nutrients or sediment conditions. However, this does not mean that such mechanisms are unimportant for seagrasses, but merely indicates that they are at least not sufficiently generic in the temperate systems that we studied to be identified by our analyses.

In our analyses, we used total nitrogen, total phosphorus and sediment grain size rather than for instance surface water chlorophyll A and suspended matter concentrations. Although these variables are more direct indicators of light conditions, they would be more circumstantial with regard to the ecosystem engineering mechanisms with which seagrass can modify its surroundings, i.e. reduction of nutrient levels [5], [19] and trapping and stabilization of silt [6], [17], [23]. Also, our choice to sample sediments outside the seagrass meadows instead of inside was based on a similar reasoning. Obviously, sediments inside the meadows are generally more silty than outside, illustrating the potential of seagrasses to trap and stabilize small particles. However, in contrast to bare sediments, these trapped particles hardly erode and therefore do not influence suspended sediment concentrations anymore [14], [17], [18], [23]. Thus, to influence light conditions, ecosystem engineering by seagrasses has to be strong enough to modify sediment grain size distribution outside the meadow by trapping silt particles inside.

Although our analysis provides a strong indication for a positive feedback in *Z. marina* ecosystems, it does not offer solid proof. Notably, the specification search yielded two other models without this feedback that were able to explain a considerable amount of variance in light conditions and seagrass density. However, from our results it seems hard to argue that the highly significant causal relation from seagrass to sediment grain size should be drawn in the opposite direction. Model 2 demonstrates that including sediment as explanatory variable for seagrass improves the fit to seagrass density by only 2.0 (model 1) to 3.5% (model 3), while the other way around seagrass is able to explain over 33% of the variance in sediment D10. Moreover, the difference in AIC between model 1 and the other two models is well over 3 (see table 2), indicating that there is considerably less support for model 2 and 3 compared to model 1 [30].

Structural equation modeling proves to be a useful tool for identifying feedback loops in complex systems like ecosystems. However, the method will not be able to determine all feedback loops, as there are problems that are not “identifiable” (i.e. cannot be solved) [31]. The model should preferably include one or more “instrumental variables” (i.e. a variable outside the feedback loop that affects only one of the variables inside the loop) or otherwise the feedback loop should at least be indirect (i.e. involving more than 2 variables). Obviously, the dataset should also have enough variation in conditions to determine the correlations accurately. Here, we have an indirect loop (involving 3 variables), an instrumental variable (nutrients influencing light conditions) and a dataset that includes a wide range of environmental conditions.

In conclusion, we demonstrate that by using structural equation modeling, it is possible to identify positive feedbacks in complex systems such as ecosystems. Additionally the method can provide a mechanistic explanation and quantify its importance relative to other environmental factors. However, we stress here that SEM only provides indications for positive feedback mechanisms, not for alternative stable states nor does it offer any indication for imminent shifts. Therefore, this method should preferably be used in concert with other methods that can hint for bistability like bimodality or hysteresis [2] which can provide indications for catastrophic collapse, such as critical slowing down or spatial patterns [32].
